# Effects of pulmonary rehabilitation on functional and psychological parameters in post-acute sequelae of SARS-CoV-2 infection (PASC) patients

**DOI:** 10.1186/s12890-024-03047-0

**Published:** 2024-05-14

**Authors:** Adeel Nasrullah, Shiza Virk, Anam Javed, Aaisha Shah, Deeksha Ramanujam, Alisha Sharma, Laura Gutierrez, Kevin Nauer, Mindy Maggio, Yue Yin, Yousaf Bajwa, Tariq Cheema, Briana Disilvio

**Affiliations:** 1 Division of Pulmonary and Critical Care, Allegheny Health Network, Pittsburgh, PA USA; 2 Department of Internal Medicine, Allegheny Health Network, Pittsburgh, PA USA; 3 Lifeline Physical Therapy, Warrendale, PA USA; 4 Allegheny-Singer Research Institute, Allegheny Health Network, Pittsburgh, PA USA; 5 University of Pittsburgh, Pittsburgh, PA USA

**Keywords:** Post-acute Sequelae of SARS-CoV-2 infection, Long COVID, Post-acute COVID-19, Pulmonary Rehabilitation

## Abstract

**Background:**

COVID-19 survivors may develop long-term symptoms of fatigue, dyspnea, mental health issues, and functional limitations: a condition termed post-acute sequelae of COVID-19 (PASC). Pulmonary rehabilitation (PR) is a recommended treatment for PASC; however, there is a lack of data regarding PR’s effect on multiple health indices and the factors that influence patient outcomes. The aim of our study is to evaluate the impact of pulmonary rehabilitation on functional and psychological parameters in patients diagnosed with Post-Acute Sequelae of SARS-CoV-2 Infection (PASC), thereby offering insights into the efficacy of such interventions in improving the quality of life and clinical outcomes for these individuals.

**Methods:**

We extracted patient demographic, comorbidity, and outcome data from Allegheny Health Network’s electronic medical records. Functionality test results were compared before and after PR, including 6-minute walk test (6MWT), chair rise repetitions (CR reps), timed up and go test (TUG), gait speed (Rehab gait), modified medical research council scale (MMRC), shortness of breath questionnaire (SOBQ), hospital anxiety and depression scale (HADS) and chronic obstructive pulmonary disease assessment test (CAT) scores. Multiple regression analysis was done to evaluate the effect of comorbidities and patient factors on patient responses to PR.

**Results:**

The 55 patients included in this study had a mean time of 4 months between the initial COVID-19 diagnosis and the subsequent PASC diagnosis. Following pulmonary rehabilitation (PR), significant improvements were observed across various metrics. The distance covered in the 6-minute walk test (6MWT) increased markedly from a pre-rehabilitation average of 895 feet (SD 290) to 1,300 feet (SD 335) post-rehabilitation, with a mean change of 405 feet (95% CI [333, 477]). Chair rise repetitions (CR reps) saw an increase from 9 (SD 3) reps to 13 (SD 3) reps, with a change of 4 reps (95% CI [3.7, 4.9]). The timed up and go test (TUG) time decreased significantly from 13 s (SD 5) to 10 s (SD 2), reflecting a mean reduction of 3 s (95% CI [-4.5, -2.5]). Rehabilitation gait speed improved from 1.0 m/s to 1.3 m/s, changing by 0.3 m/s (95% CI [0.2, 0.3]). The Modified Medical Research Council (MMRC) dyspnea scale showed a notable decrease from a mean of 2 (SD 1) to 1 (SD 1), a change of -1 (95% CI [-1.5, -1]). The Shortness of Breath Questionnaire (SOBQ) scores reduced significantly from 51 (SD 21) to 22 (SD 18), with a change of -29 (95% CI [-34, -23]). The Hospital Anxiety and Depression Scale (HADS) scores decreased from 11 (SD 7) to 8 (SD 7), a reduction of -4 (95% CI [-5, -2]). Lastly, the Chronic Obstructive Pulmonary Disease (COPD) Assessment Test (CAT) scores significantly dropped from 18 (SD 7) to 9 (SD 7), changing by -10 (95% CI [-11, -8]). However, the presence of hypertension, diabetes, chronic lung diseases, outpatient status, and receipt of specific pharmacologic treatments (decadron, decadron + remdesivir, and decadron + remdesivir + tocilizumab) were identified as factors associated with a poor response to PR.

**Conclusion:**

Our study supports PR as an integrated model of care for PASC patients to improve several physical and mental health indices. The long-term effects of PR on patients’ functional status should be investigated in the future.

## Background

The COVID-19 pandemic, caused by the SARS-CoV-2 virus, has had an immense impact on public health, posing significant challenges for healthcare systems worldwide. Among the most substantial of these challenges are the long-term consequences experienced by a significant number of COVID-19 survivors, known as post-acute sequelae of SARS-CoV-2 infection (PASC) or “Long COVID” [1]. Both the Centers for Disease Control and Prevention and the World Health Organization have acknowledged PASC as a condition persisting after SARS-CoV-2 infection, and it is estimated to have a prevalence of approximately 5% in non-hospitalized patients and almost 80% in hospitalized patients [2, 3]. Temporal definitions for PASC vary, but it is typically recognized as symptoms persisting from three to 24 weeks after the initial infection. A comprehensive meta-analysis also reported an average symptom cluster duration of nine months in hospitalized patients versus four months in non-hospitalized patients [4]. PASC includes a broad range of symptoms and health complications, with persistent respiratory difficulties and functional limitations being among the most common and debilitating ones.

The pathological mechanisms contributing to PASC symptoms are multifaceted, encompassing the effects of the SARS-CoV-2 infection itself, virus reactivation, autoimmunity, incomplete recovery from acute organ injury, effects of treatment (mechanical ventilation, steroids), and exacerbation of pre-existing conditions, such as diabetes mellitus (DM), chronic kidney disease (CKD) and congestive heart failure (CHF) [5]. While acute COVID-19 primarily impacts respiratory function, PASC affects multiple systems, leading to symptoms that include severe fatigue, breathlessness, and impaired physical functionality [6]. Pulmonary damage and reduced diffusion capacity following acute lung injury may also make significant contributions to long-term functional limitations [7]. These persistent symptoms have the potential to significantly compromise the quality of life (QOL) of PASC patients, contributing to a decline in physical performance, autonomy, and mental health.

Currently, there is no medication that has been proven effective for the treatment of PASC. In response to this growing health concern, there has been an increasing interest in the potential of pulmonary rehabilitation (PR) as a therapeutic intervention for individuals with PASC. Considering the potential benefit with limited associated risk, the European Respiratory Society and American Thoracic Society task force has recommended early rehabilitation at the bedside to mitigate the debilitating effects associated with COVID-19 [8]. PR is a multidisciplinary approach to care that has been widely recognized for its benefits in managing chronic respiratory diseases (chronic obstructive lung disease and interstitial lung disease [ILD]), including improvements in dyspnea, physical functionality, mental health, and overall QOL [7, 9, 10]. These benefits even extend to patients with mild COVID-19 disease, highlighting the importance of exercise training for individuals at risk of long-term sequelae [11].

Recently, several observational studies and clinical trials have demonstrated the efficacy of both home and supervised rehabilitation exercises in managing PASC [12]. Notably, the RECOVERY trial, involving 80 non-hospitalized adults diagnosed with post-COVID-19 syndrome, found supervised multicomponent exercise programs and endurance training to be significantly more effective in symptom reduction than respiratory therapy alone, with or without at-home inspiratory muscle training. Preliminary studies have also indicated that PR may enhance exercise capacity, reduce fatigue, and improve pulmonary functions in PASC patients [1, 13, 14].

Our analysis of recent literature reveals that pulmonary rehabilitation (PR) holds significant promise for improving the functional and psychological outcomes of patients with post-acute sequelae of SARS-CoV-2 infection (PASC). Evidence from a rapid review and a systematic review and meta-analysis consistently demonstrate PR’s effectiveness in enhancing exercise capacity, with notable improvements in the 6-minute walk test (6MWT) across patient populations recovering from COVID-19 [1, 15]. These findings are particularly relevant for our study, underscoring the potential of PR in the integrated model of care for PASC patients. While improvements in pulmonary function, dyspnea, and quality of life are reported, the data suggest variability in outcomes, indicating a need for tailored PR programs that consider individual patient conditions and the multifaceted nature of PASC. Our study aims to further elucidate the role of PR in the rehabilitation of PASC patients, contributing to the growing body of evidence supporting PR as a critical component of post-COVID-19 care and recovery. Moreover, an understanding of the optimal protocols for PR in the context of PASC, as well as its long-term effects and cost-effectiveness, require further exploration.

Therefore, our study aims to evaluate the impact of PR on mobility, dyspnea scales, functionality, and mental health in patients diagnosed with PASC at our tertiary care center. We found that PR has potential as a therapeutic intervention for PASC, thereby enhancing our understanding of how to best support the recovery and well-being of COVID-19 survivors.

## Methods

This retrospective study used electronic medical records from Allegheny Health Network (AHN) as the primary data source. The patient population included in this study consisted of individuals whose information was already shared as part of the existing vendor contract between AHN and the Lifeline PR Program (Lifeline) when a patient is referred for PR.

### Study population

This study included patients who were evaluated at an AHN clinic (69% seen at post-COVID clinic and 21% from other clinics), determined to have PASC based on European Respiratory Society guidelines [16], and subsequently referred to Lifeline. To be included in the study, patients were required to be ≥ 18 years old and diagnosed with “History of COVID-19 infection”, “Post-COVID syndrome”, “Long COVID”, or any synonymous diagnoses. There were no explicit exclusion criteria.

### Pulmonary rehabilitation program

Our study partnered with Lifeline Physical Therapy and Pulmonary Rehabilitation Centers to deliver a comprehensive post-COVID-19 recovery program. The program consists of an orientation session, initial assessments by physical and respiratory therapists, and a risk assessment to develop a personalized care plan. It emphasizes a multidisciplinary approach, incorporating goals to improve activities of daily living, functional abilities, strength, endurance, and self-management skills. The rehabilitation schedule typically spans three months, with sessions held two to three times a week, available through in-facility, telehealth, and a hybrid mode. The program concludes with an evaluation to assess progress and provide patients with ongoing recommendations and a certificate of completion, ensuring a tailored and patient-centric rehabilitation journey.

### Data extraction

After identifying the study population, specific data were extracted from the patients’ charts. Patient demographics (age, gender, body mass index [BMI], race, and insurance status), lifestyle factors and history (smoking, alcohol use, drug use), comorbidities, hospitalization details, and COVID-19 treatment information were collected. Our study utilized de-identified patient data as part of a quality improvement (QI) initiative at Allegheny Health Network (AHN). Due to the nature of the study—focusing on de-identified data for quality improvement purposes—formal patient consent for data collection was not required. This approach aligns with standard practices for QI studies, ensuring patient privacy and data protection while allowing for the analysis of clinical outcomes. These data were analyzed to investigate the effect and influence each factor had on the patient’s pulmonary health, functional status, and PR outcomes.

### Study outcomes

The primary study outcomes were changes in the patient’s health indices and test results before and after PR. A detailed examination of diagnostic and functional test results was conducted, including imaging, pulmonary function test, and cardiopulmonary exercise testing results. We assessed changes in patients’ results for the 6MWT, TUG, CR reps, balance test, and gait speed (Rehab gait). Additionally, we evaluated the differences in modified medical research council dyspnea scale (MMRC), shortness of breath questionnaire (SOBQ), hospital anxiety and depression scale (HADS), chronic obstructive pulmonary disease assessment test (CAT), and patient health questionnaire-9 (PHQ-9) scores before and after PR. These data were used to quantify the effectiveness of PR for each PASC patient.

### Statistical analysis

We used descriptive statistics to present our results, providing the mean and standard deviation (SD) for continuous variables and the frequencies and percentages for categorical variables. Based on the data distribution and characteristics, we used the Paired T-test and the Wilcoxon signed-rank test to assess the mean differences between primary variables before and after PR. Given the high number of potential predictors, we used a stepwise selection process to identify significant predictors that influence the value change in primary variables from pre- to post-rehabilitation. PR outcomes were calculated as the post-rehabilitation value minus the pre-rehabilitation value. The threshold for statistical significance was set at an alpha level of 0.05. All statistical analyses were performed using SAS software, version 9.4.

### Patient demographics

We identified 55 patients diagnosed with PASC; there was a mean time of 4 months between the initial COVID-19 diagnosis and the PASC diagnosis. Baseline characteristics can be found in Table 1.

**Table 1:** Demographics and comorbidities of patients with post-acute sequelae of SARS-CoV-2 infection

Variable		Mean (SD)
Age		58 (14)
Body mass index		32 (8)
Number of COVID-19 infections		1 (0)
Hospitalization duration		8 (11)
		**Frequency (%)**
Gender	Male	22 (40)
	Female	33 (60)
Race	Caucasian	51 (93)
	African American	4 (7)
Insurance	Medicare	15 (27)
	Medicaid	1 (2)
	Private	39 (71)
Smoking	Never	36 (65)
	Active	51 (93)
	Past smoker	1 (2)
Alcohol use	Never	1 (2)
	Moderate*	2 (4)
	Unhealthy**	53 (96)
	Not documented	2 (4)
Drug use	Never	36 (65)
	Active	19 (35)
	Past use	42 (76)
	Not documented	13 (24)
Congestive Heart Failure	No	53 (96)
	Yes	2 (4)
Obstructive Sleep Apnea	No	42 (76)
	Yes	13 (24)
Asthma	No	29 (53)
	Yes	26 (47)
Pulmonary Hypertension	No	51 (93)
	Yes	4 (7)
Diabetes Mellitus	No	48 (87)
	Yes	7 (13)
Hypertension	No	55 (100)
	Yes	51 (93)
Interstitial Lung Disease	No	1 (2)
	Yes	1 (2)
Chronic Kidney Disease	No	2 (4)
	Yes	53 (96)
Liver Cirrhosis	No	2 (4)
Dialysis dependent	No	55 (100)
Human Immunodeficiency Virus Infection	No	55 (100)
Malignancy	Never	48 (87)
	Active	2 (4)
	Remission	5 (9)
Vascular disease	No	54 (98)
	Yes	1 (2)
Depression	No	32 (58)
	Yes	23 (42)
Transplant	Never	55 (100)
PFT interpretation	Normal	5 (9)
	Obstructive disease	3 (5)
	Restrictive	2 (4)
	Isolated DLCO decrement	4 (7)
	Not available	41 (75)

* Females: <7 drinks/week; males: <14 drinks/week

**Females: >7 drinks/week; males: >14 drinks/week

PFT: pulmonary function test, DLCO: diffusing capacity of the lungs for carbon monoxide

### Comorbidities

Patients in the study population reported different comorbidities affecting different organ systems. The most common comorbidities in the study population were hypertension (47%, *n* = 26) and depression (42%, *n* = 23). Further details are included in Table 1.

### COVID-19 course and therapies

For treatment of COVID-19, 37% (*n* = 20) of the patients were managed as outpatients, while 63% (*n* = 34) were hospitalized for their initial infection. We were unable to determine the treatment course for one patient. For outpatients, 11% (*n* = 6) of patients received monoclonal antibodies, 6% (*n* = 3) received inhaled corticosteroids (ICS), and 83% (*n* = 45) received no therapy. Among those hospitalized, 35% (*n* = 19) of patients received no therapy, 13% (*n* = 7) received decadron, 37% (*n* = 20) received decadron and remdesivir, and 15% (*n* = 8) received decadron, remdesivir, and tocilizumab or baricitinib simultaneously.

Respiratory system support occurred through several modalities. In total, 39% (*n* = 21) of patients were managed with nasal cannula, of which (*n* = 18) of patients were either not on oxygen or required more than 2 L oxygen from their baseline oxygen requirement, 7% (*n* = 4) managed with Optiflow, 15% (*n* = 8) managed with non-invasive ventilation (NIV), and 6% (*n* = 3) intubated. In terms of inhaler use, 22% (*n* = 12) of patients used short-acting beta-agonists (SABA), 4% (*n* = 2) used SABA with ICS (SABA-ICS), 27% (*n* = 15) used long-acting beta-agonists with ICS (LABA-ICS), 7% (*n* = 4) used long-acting muscarinic antagonists with LABA and ICS (LAMA-LABA-ICS), and 40% did not use an inhaler.

Post-COVID-19 transthoracic echocardiogram (TTE) results were not available for 56% (*n* = 31) of patients, but 36% (*n* = 20) of patients had a normal TTE, 4% (*n* = 2) showed new systolic dysfunction, and 4% (*n* = 2) showed new diastolic dysfunction. Table 2 shows COVID-19 therapies received by this cohort.

**Table 2:** COVID-19 treatments for patients with post-acute sequelae of SARS-CoV-2 infection

COVID treatment types	Specific treatment	Frequency (%)
COVID-19 course	Outpatient managed	20 (37)
	Hospitalized	34 (63)
Outpatient therapies	None	45 (83)
	Monoclonal antibodies	6 (11)
	ICS	3 (5)
Inpatient therapies	None	19 (35)
	Decadron	7 (13)
	Decadron + remdesivir	20 (37)
	Decadron + remdesivir + tocilizumab or baricitinib	8 (15)
Supplemental oxygen	None or at baseline	18 (33)
	Nasal canula	21 (39)
	Optiflow	4 (7)
	NIV	8 (15)
	Intubation	3 (5)
Transthoracic Echocardiogram Post- COVID-19	Not available	31 (56)
	Normal	20 (36)
	New systolic dysfunction	2 (3)
	New diastolic dysfunction	2 (3)
Inhalers	None	22 (40)
	SABA	12 (22)
	SABA-ICS	2 (3)
	LABA-ICS	15 (27)
	LAMA-LABA-ICS	4 (7)

* ICS: inhaled corticosteroids; NIV: non-invasive ventilation; SABA: short-acting beta-agonists; SABA-ICS: short-acting beta-agonists with inhaled corticosteroids; LABA: long-acting beta-agonists with inhaled corticosteroids; LAMA-LABA-ICS: long-acting muscarinic antagonists with long-acting beta-agonists and inhaled corticosteroids

### Effect of pulmonary rehabilitation on various functional and psychological tests

Table 3 shows pre and post pulmonary rehabilitation improvement in various metrics of functional and psychological testing. When comparing patient outcomes and functional test results pre- and post-PR, overall, both improved following rehabilitation. The distance (6MWD) covered by patients during the 6MWT significantly improved from a pre-rehabilitation mean of 895 (± 290) ft to a post-rehabilitation mean of 1300 (± 335) ft by changing 405, 95% CI [333, 477], with a T-value of 11.35 and a p-value < 0.0001. The number of patient CR reps significantly increased from an average of 8.83 (± 3) reps pre-rehabilitation to 13.13 (± 3) reps post-rehabilitation by changing 4.31, 95% CI [3.71, 4.90]. The T-value for this comparison was 14.53, with a p-value < 0.0001. Patient Rehab gait increased from an average of 1.03 (± 0.24) m/s to 1.30 (± 0.18) m/s post-rehabilitation by changing 0.27, 95% [0.21, 0.33], with a T-value of 8.62 and a p-value < 0.0001. TUG outcomes demonstrated significant improvement in patients after PR. Patients’ average TUG time decreased from 12.76 (± 5) seconds to 9.50 (± 2.41) seconds post-rehabilitation by changing − 3.50, 95% CI [-4.53, -2.47], with a T-value of -6.93 and a p-value < 0.0001. However, patients with DM or ILD, outpatients during COVID-19 treatment, and patients that received specific inpatient therapies (decadron, decadron + remdesivir, decadron + remdesivir + tocilizumab) showed an increase in time taken to complete the TUG test.

**Table 3:** Comparison between pre- and post-pulmonary rehabilitation for patients with post-acute sequelae of SARS-CoV-2 infection

Variable	Comparison		Mean Change[95% CI]	T-value/S-value	*P*-value
	Pre	Post			
6MWT	895(290)	1300(335)	405[333, 477]	11.35	< 0.0001
CR reps	8.83(2.78)	13.13(3)	4.31[3.71, 4.90]	14.53	< 0.0001
TUG	12.76(4.79)	9.50(2.41)	-3.50[-4.53, -2.47]	-6.93	< 0.0001
Rehab gait	1.03(0.24)	1.30(0.18)	0.27[0.21, 0.33]	8.62	< 0.0001
MMRC	2.13(1)	0.89(1)	-1.20[-1.46, -0.95]	-9.39	< 0.0001
SOBQ	50.64(21)	21.82(18)	-28.55[-34.47, -22.62]	-9.72	< 0.0001
HADS	11.36(7)	7.78(7)	-3.59[-5.04, -2.14]	-4.99	< 0.0001
CAT	18.21(7)	8.60(7)	-9.66[-11.29, -8.03]	-11.98	< 0.0001
PHQ	1.71(4)	2.33(5)	0.63[-1.13, 2.40]	4	0.7402

6MWT: 6 min Walk Test; CR reps: Chair Rise repetitions; TUG: Timed Up and Go Test; Rehab gait: Rehabilitation Gait; MMRC: Modified Medical Research Council; SOBQ: Shortness of Breath Questionnaire; HADS: Hospital Anxiety and Depression Scale; CAT: COPD Assessment Test; PHQ: Patient Health Questionnaire

PR was generally associated with better scores on outcome tests, but certain factors were associated with worse outcomes. The MMRC dyspnea scale significantly decreased from a mean of 2.13 (± 1) to 0.89 (± 1) after rehabilitation by changing − 1.20, 95% CI [-1.46, -0.95], with a T-value − 9.39 and a p-value < 0.0001. However, being Caucasian or having DM, HTN, or CKD were associated with higher scores post-PR compared to pre-PR scores. The SOBQ score reduced substantially from a pre-rehabilitation mean of 50.64 (± 21) to 21.82 (± 18) post-rehabilitation by changing − 28.55, 95% CI [-34.47, -22.62], with a T-value of -9.72 and a p-value < 0.0001. While the SOBQ score showed a significant reduction post-rehabilitation, the use of antibody treatment and ICS outpatient therapies were linked with increased scores. The HADS showed a significant reduction from a mean of 11.36 (± 7) pre-rehabilitation to 7.78 (± 7) post-rehabilitation by changing − 3.59, 95% CI [-5.04, -2.14]. The T-value for this comparison was − 4.99 with a p-value < 0.0001. However, having CHF, ILD, and receiving specific inpatient therapies (decadron, decadron + remdesivir, decadron + remdesivir + tocilizumab) were associated with higher HADS scores. The CAT score, a measure of the impact of chronic obstructive pulmonary disease (COPD) on a person’s life, significantly decreased from an average of 18.21 (± 7) pre-rehabilitation to 8.60 (± 6.60) post-rehabilitation by changing − 9.66, 95% CI [-11.29, -8.03], with a T-value of -11.98 and a p-value < 0.0001. However, patients with CHF, obstructive sleep apnea, those who received antibody outpatient therapy, or those with multiple COVID-19 diagnoses had higher CAT scores post-PR than pre-PR.

Patient PHQ-9 scores increased slightly from 1.71 (± 4) pre-rehabilitation to 2.33 (± 5) post-rehabilitation, but this was not statistically significant, with an S-value of 4 and a p-value of 0.740. Finally, although the PHQ-9 score showed the slight increase post-rehabilitation, active alcohol use, the presence of CKD, receiving specific inpatient therapies (decadron, decadron + remdesivir, decadron + remdesivir + tocilizumab), and having multiple COVID-19 diagnoses were associated with higher scores. Table 4 shows regression analysis of changes from pre- to post-pulmonary rehabilitation for patients with post-acute sequelae of SARS-CoV-2 infection.

**Table 4:** Regression results on changes from pre- to post-pulmonary rehabilitation for patients with post-acute sequelae of SARS-CoV-2 infection

Outcome	Predictor		Estimate	T-value	*P*-value
6MWT	Hypertension	No	Reference	-	-
		Yes	-134.20	-2.17	0.0348
CR reps	Gender	Female	Reference	-	-
		Male	-1.18	-2.06	0.0455
	Race	African American	Reference	-	-
		Caucasian	-2.05	-2.00	0.0509
TUG	Diabetes Mellitus	No	Reference	-	-
		Yes	-2.93	-3.63	0.0012
	Interstitial Lung Disease	No	Reference	-	-
		Yes	-2.37	-2.13	0.0422
	COVID course	Hospitalized	Reference	-	-
		Outpatient	-6.36	-5.27	< 0.0001
	Inpatient therapies	None	Reference	-	-
		Decadron	-4.94	-3.66	0.0011
		Decadron + remdesivir	-4.01	-3.07	0.0048
		Decadron + remdesivir + tocilizumab	-3.86	-2.38	0.0248
MMRC	Race	African American	Reference	-	-
		Caucasian	0.77	1.96	0.0570
	Diabetes Mellitus	No	Reference	-	-
		Yes	-0.80	-3.25	0.0024
	Hypertension	No	Reference	-	-
		Yes	0.81	3.74	0.0006
	Chronic Kidney Disease	No	Reference	-	-
		Yes	-0.94	-3.23	0.0026
SOBQ	Outpatient therapies	None	Reference	-	-
		Antibody	-10.39	-1.26	0.2155
		ICS	-58.59	-3.34	0.0018
HADS	Chronic Heart Failure	No	Reference	-	-
		Yes	-9.50	-2.35	0.0243
	Interstitial Lung Disease	No	Reference	-	-
		Yes	4.38	1.76	0.0863
	Inpatient therapies	None	Reference	-	-
		Decadron	5.67	2.97	0.0052
		Decadron + remdesivir	-0.21	-0.15	0.8854
		Decadron + remdesivir + tocilizumab	2.90	1.42	0.1627
CAT	Congestive Heart Failure	No	Reference	-	-
		Yes	-9.53	-2.52	0.0161
	Obstructive Sleep Apnea	No	Reference	-	-
		Yes	2.09	1.76	0.0871
	Outpatient Therapies	None	Reference	-	-
		Antibody	-6.02	-3.37	0.0018
		ICS	-2.53	-0.67	0.5072
	Number of COVID diagnoses		-5.84	-3.96	0.0003
PHQ	Alcohol use	Never	Reference	-	-
		Active	-3.89	-3.27	0.0037
		Past smoker	0.04	0.01	0.9897
	Chronic Kidney Disease	No	Reference	-	-
		Yes	3.06	1.74	0.0964
	Inpatient therapies	None	Reference	-	-
		Decadron	12.91	4.63	0.0001
		Decadron + remdesivir	-3.16	-2.31	0.0310
		Decadron + remdesivir + tocilizumab	-4.08	-2.19	0.0403
	Number of COVID diagnoses		-5.03	-3.14	0.0049

6MWT: 6 min Walk Test; CR reps: Chair Rise repetitions; TUG: Timed Up and Go test; MMRC: Modified Medical Research Council; SOBQ: Shortness of Breath Questionnaire; HADS: Hospital Anxiety and Depression Scale; CAT: COPD Assessment Test; PHQ: Patient Health Questionnaire; ICS: inhaled corticosteroids

## Discussion

PR involves exercise training, education, and behavioral and lifestyle rehabilitation and is a promising intervention for facilitating recovery and enhancing QOL in patients with long COVID-19 [17]. Our observational study findings strongly support the beneficial effects of PR for PASC patients with varying degrees of disease severity. There were significant improvements in mobility, dyspnea scales, functionality, and mental health.

Our data provide robust evidence supporting the positive effect of PR on physical health in those suffering with long COVID-19. Despite their age and high baseline 6MWD, participants still improved their walking distance by nearly two standard deviations, which is above the average minimal clinically important difference (MCID) for the test [18]. In a similar study conducted with 64 patients, 70% of participants 6MWD improved nearly twice the MCID from baseline (63 +/- 48) after 6 weeks of rehabilitation [19]. Similarly, we saw a significant increase in other indices above their MCID; CR reps improved (9 to 13 reps, *p* < 0.001), TUG test time (12.76 to 9.50 s, *p* < 0.0001) and gait speed (1.03 m/s to 1.30 m/s, *p* < 0.0001) [20–22]. While the patients were still symptomatic, their QOL improved, and the improvements in these health indices after PR support it as an effective treatment for the physical symptoms of PASC. This is consistent with the improvements seen in populations of former critically ill patients who are recovering from acute respiratory distress syndrome and those with post-intensive care syndrome who experience slow recovery, long-term cognitive impairment, and functional disability [23, 24].

We also found that PR is associated with improved mental outcomes. Our study showed significant improvement in PASC patient HADS scores after PR, with only minor, statistically insignificant worsening in mean PHQ-9 scores. The benefits of PR for mental health have been confirmed in patients with several health conditions, such as COPD and asthma, and similar positive effects are being seen in patients with PASC [10, 25, 26]. A prospective study of 50 patients showed marked improvements in QOL for those with severe/critical COVID-19 after rehabilitation, particularly in their SF-36 mental component scores [14]. In our study, CHF, ILD, and receiving specific inpatient therapies were associated with higher HADS scores, suggesting a possible increase in psychological distress and resistance to improved anxiety and depression in patients with these factors. Active alcohol use, CKD, receiving specific inpatient therapies, and having multiple SARS-CoV-2 infections were also associated with higher PHQ-9 scores, which suggests that these factors may also worsen psychological health.

Beyond the stress induced by COVID-19 itself, public health measures, such as mass confinement and the ensuing financial losses, have substantially increased emotional distress and psychiatric illness risk. Our study showed significant improvement in HADS scores post-rehabilitation which is consistent with prior reported literature. A systematic review showed elevated rates of anxiety (6–51%), depression (14–48%), post-traumatic stress disorder (7–54%), and psychological stress (34–38%) in the general population during the pandemic [27]. Post-recovery, COVID-19 patients commonly exhibit long-term anxiety and depression symptoms [28]. A 2021 study tracking 1050 discharged COVID-19 patients revealed that those with positive PHQ-2 and trauma screening questionnaire (TSQ) scores were more likely to experience persistent dyspnea, myalgia, anorexia, and confusion at a 9-week follow-up (PHQ-2 80% vs. 41.8%, TSQ 89% vs. 43%, *p* < 0.001) [29].

Dyspnea, one of the most prevalent symptoms of PASC, is most likely multifactorial [30]. This symptom is common, persistent, and has a negative impact on patient QOL, often leading to poor sleep and low mood [31]. Our study highlights a significant decrease in MMRC, SOBQ, and CAT scores beyond their MCID. This is corroborated by a meta-analysis published by Ahmed et al. [32], which included eight randomized controlled trials with 449 participants. Their findings revealed that PR significantly improves dyspnea and exercise capacity [32]. However, in our study, antibody treatment and ICS were linked to increased SOBQ scores despite the overall SOBQ score reduction. Our study results confirm existing research showing that PR may improve dyspnea in patients with long COVID.

Comorbidities and risk factors like hypertension, DM, and previous SARS-CoV-2 infection have been shown to influence recovery rates, presenting challenges to patient mobility and psychological health and causing dyspnea [33, 34]. More specifically, conditions like hypertension, DM, COPD, CHF, ILD, multiple COVID-19 diagnoses, obstructive sleep apnea, and inpatient and outpatient COVID-specific therapies are associated with slower recovery and may limit improvement in mobility, dyspnea and psychological health [35]. To our knowledge, our study presents the first insight into the effects of these factors on PASC and PR outcomes. The results of this study have unveiled potential avenues for future research on complex interactions between each of these risk factors to identify pathological mechanisms and ultimately restore a patient’s physical and psychological health after COVID-19.

It is important to note that our study cohort was predominantly Caucasian, whereas other races such as African Americans and Hispanics have been shown to have a higher risk of contracting COVID-19 [36, 37]. This draws attention to the socioeconomic, ethnic, and health disparities associated with chronic respiratory diseases that may pose barriers to PR [38, 39]. The COVID-19 pandemic has greatly amplified and exposed the existing inequalities and social determinants of health in patients with chronic respiratory disease [40]. These disparities may directly impact the uptake, attendance, and completion of PR and subsequent recovery from PASC. Clinicians and policymakers must consider these systemic differences in race and socioeconomic factors to optimize rehabilitation care for a diverse population. Our study’s findings indicate a distinct variation in the efficacy of PR for PASC when comparing outcomes between male and female participants, with males exhibiting less improvement than females. This observation is particularly noteworthy as existing literature on PR outcomes for PASC patients, including comprehensive reviews and meta-analyses, generally does not segregate data based on gender. The majority of prior studies have focused on evaluating the overall effectiveness of various PR approaches—ranging from traditional methods to innovative solutions like virtual reality and tele-rehabilitation—without explicitly examining gender as a variable influencing rehabilitation outcomes. This gap suggests a significant opportunity for future research to delve into gender differences in PR efficacy. Such targeted analysis could unveil critical insights into personalized rehabilitation strategies, ensuring optimized outcomes for all patients recovering from COVID-19.

Our study illuminates the need for continued longitudinal research on post-COVID-19 recovery, focusing on the long-term impacts of personalized PR for patients with PASC. It underscores the importance of integrating mental health services within these programs and investigating the efficacy of remote rehabilitation methods. There is potential benefit in exploring a proactive approach and starting rehabilitation immediately post-recovery. This study highlights a broader focus on QOL beyond clinical outcomes and the need to address health disparities in accessing PR. Lastly, an understanding of the cost-effectiveness of these interventions is crucial for resource allocation and policy making. Hence, future research may shed more light on the optimal management of patients with PASC, thereby reducing disease burden, streamlining health service utilization, and enhancing QOL.

### Limitations and strengths

Our retrospective study offers significant insights but faces several limitations. As an observational study, it lacks randomization and a control group, which are crucial for reducing biases and bolstering result validity. The limited sample size potentially impacts statistical power and the robustness of the study, limiting the wider applicability of our findings to all PASC patients. The study does not clearly distinguish improvements from natural recovery versus those from PR intervention. It also lacks long-term follow-up to assess lasting effects of the rehabilitation. The potential overlap of chronic fatigue syndrome with PASC is another limitation. Despite these constraints, the study’s pragmatic approach in a real-world setting is an asset, and the average follow-up period of 3.8 months post-rehabilitation allows for a reasonable evaluation of outcomes. However, further randomized control trials with larger samples and longer-term follow-up are necessary to confirm and expand these findings.

## Conclusions

In summary, our study supports PR as an integrated model of care for patients with PASC to improve several indices of both physical and mental health. Studying how and if PR promotes long-term improvement in patient functional status should be the focus of future studies and trials.

## Data Availability

The data that supports the findings of this study are available on request from the corresponding author. AN. This is in accordance to ensure individuals’ privacy under the European General Data Protection Regulation.

## Abbreviations

6MWT: 6 min walk test
6MWD: 6 min walk distance
AHN: Allegheny Health Network
BMI: Body mass index
CAT: Chronic obstructive pulmonary disease assessment test
CHF: Congestive heart failure
CKD: Chronic kidney disease
COPD: Chronic obstructive pulmonary disease
CR: Chair rise repetitions
DLCO: Diffusing capacity of the lungs for carbon monoxide
DM: Diabetes mellitus
HADS: Hospital anxiety and depression scale
HSPSO: Health System Publication Support Office
ICS: Inhaled corticosteroids
ILD: Interstitial lung disease
IRB: Institutional Review Board
LABA: Long-acting beta-agonists
MCID: Minimal clinically important difference
MMRC: Modified medical research council
NIV: Non-invasive ventilation
PASC: Post-acute sequelae of COVID-19
PHQ: Patient health questionnaire
PR: Pulmonary rehabilitation
QOL: Quality of life
SABA: Short-acting beta-agonists
SD: Standard deviation
SOBQ: Shortness of breath questionnaire
TSQ: Trauma screening questionnaire
TUG: Timed up and go
TTE: Transthoracic echocardiogram
